# Reciprocity in Licensure Once More

**Published:** 1903-01

**Authors:** 


### Reciprocity in Licensure Once More.

THE subject of interstate reciprocity in medical license has been discussed in these columns on many occasions, and we feel that it is almost unnecessary to return to the subject until we can offer something new to our readers relating to this vexed question. The journals have been printing considerable material of late that has dealt with many features of the proposition, much of which has missed its essential points, but we find an editorial article in the *Boston Medical and Surgical Journal* for December 18, 1902, which exhibits such a grasp of the subject as well as such a breadth of knowledge of all its bearings, that we feel justified in giving it in full, and we ask all interested to read it carefully, because it is a succinct statement of the real facts in the case, set forth in a plain, straightforward manner. It is as follows:

RECIPROCITY, OR INTERSTATE LICENSURE OF PHYSICIANS.

The impression is more or less general among medical men that a person registered as a physician in one state should, on removing to another, be recognised by the authorities therein as a qualified practitioner and licensed to practise, without being required to take another state examination, or even to pay another state registration fee. For several years this matter has received considerable attention both in the medical press and in the discussions of medical men in many of the states.

A few years ago a national organisation was effected by the examining and licensing boards of the country, in an endeavor to bring about as nearly as possible a uniformity not only of methods of procedure but of qualifications required for licensure. It was thought by the promoters of this organisation that under a uniformity of requirements by the various boards, a system of reciprocity might ultimately result, whereby a physician licensed by any recognised state board might secure a license to practise within the jurisdiction of any other board, without incurring the expense and trouble of a new examination. But in this respect, thus far, the work of this organisation has proved a disappointment. In the path of reciprocity, so to speak, many obstacles appear which perhaps at first sight do not present themselves as hindrances at all.

The examining boards in the different states are differently organised. They act under different laws, making different requirements. In a number of states, Michigan, for instance, and Colorado, Kentucky, Nebraska, Vermont and several others, a diploma from a recognised medical school is the only qualification required for licensure. In Massachusetts, New York, Pennsylvania and various other states, the laws require that every applicant shall be examined as to his qualifications to practise. And even in states requiring an examination of all, it is easy to see that standards of qualification may differ widely. Each state is naturally jealous of its own work and standards, especially its educational standards. The several states, so far as their internal affairs are concerned, are quite independent of each other. Each makes its own laws and cares for its own people in its own peculiar way, and exercises no more authority over or in the affairs of other states of the Union than does France in the affairs of Great Britain. Hence arise difficulties nearly, if not quite, insuperable in the way of medical reciprocity.

The contention sometimes heard as to the desirability of a national law governing practice is utterly futile from the fact that the national government cannot legislate at all on such matters. The constitution, the organic law of the union of the states, does not permit such interference in state affairs.

In a few states, notably New Jersey and Maine, the examining boards have provided for a limited interchange of registration certificates issued by certain states. But the "red tape" guarding this process of registration, taken with the fact that the fee of registration through interchange of certificates is double that required for registration by the examination process, is quite enough to render such a system of registration undesirable, if not altogether impracticable.

It is clearly within the authority of examining boards, as usually constituted, in testing the qualifications of applicants for registration, to give weight to the credentials a practitioner may bring from another state board or from his former peers in practice. This is the kind of reciprocity one would naturally expect and which unquestionably is regarded by all state boards. Under existing circumstances this is about as far as reciprocity is likely to go in the near future.

---

THE following resolutions were adopted by the council and Fellows of The New York State Medical Association at the annual meeting, held at the New York Academy of Medicine, October 20, 1902:

*Resolved*, That the report of the committee appointed to confer with a committee representing the Medical Society of the State of New York for the purpose of devising a plan for the union of the New York State Medical Association and the Medical Society of the State of New York, is hereby approved.

*Resolved*, That the plan presented at the joint session of the two committees by the committee representing this association, whereby, "The New York State Medical Association and the Medical Society of the State of New York be reconstituted by an act of the legislature into a state medical body, to be known as the Medical Society of the State of New York, of which all members in good standing in both bodies shall be charter members, and the reconstituted state medical body shall be the representative in this state of the American Medical Association by virtue of its acceptance of the constitution and by-laws of the American Medical Association," is hereby accepted by The New York State Medical Association as an expression of our sincere desire for a union of the medical profession in this state.

*Resolved*, That the committee is hereby continued for the purpose of coöperating with any committee from the Medical Society of the State of New York to secure a charter from the legislature at its next session in 1903, which charter shall reconstitute the two state organisations into one state body, as set forth in the preceding resolution; but if the Medical Society of the State of New York shall fail to approve of such plan of union by a charter, to be secured at the approaching session of the legislature in 1903, then this committee shall be considered as discharged and the proposition of this association withdrawn.

*Resolved*: In case this committee should find occasion to apply to the legislature at its next session for the purpose of securing the said charter it shall coöperate with the standing committee on legislation of this association.

---

THE State Department of Health now furnishes diphtheria antitoxin, prepared and tested in its antitoxin laboratory, under the following regulations: It is to be supplied for the treatment or immunisation of the following persons—namely, inmates of state institutions; inmates of other charitable institutions located in New York state; persons residing in this state unable to purchase the remedy.

Antitoxin prepared and distributed by the department is not to be sold under any circumstances, and a violation of the above prescribed rules will subject the violator to the penalty provided by section 397 of the penal code.

Physicians obtaining antitoxin for use as above noted, must agree to report the results from its use on the proper blank. Those entitled to receive the remedy under the above schedule should apply as follows: state institutions, to the state department of health, Albany; all others to the health officer of the city, town or village where they are located or reside.

---

AT A meeting of the common council of Buffalo, held December 15, 1902, a resolution was offered by Ald. Mullenhoff and passed without a dissenting vote, calling upon the Corporation Counsel to draft such an amendment to the statutory law as will prohibit treatments which are not bona fide physical treatments, and making such offences criminal acts, and that the senators and assemblymen representing Erie County be directed to use all honorable means and do all in their power to have the legislature pass the proposed amendment to the laws of this state. The resolution was preceded by several statements concerning Antonius, who has advertised himself throughout Buffalo and vicinity as the "Boy Wonder and Healer."

The foregoing action of the city legislature was prompted by the commendable course of *The Buffalo Review* in holding up to public view the fakir above referred to. It is to be hoped that the splendid efforts of the *Review* will serve to stimulate the district attorney's office to take action that will rid Buffalo of an unmitigated nuisance.

---

At a meeting of the regents of the University of the State of New York, held at Albany, December 5, 1902 the Right Rev. William Croswell Doane, Protestant Episcopal Bishop of Albany, was elected chancellor to succeed the late Anson Judd Upson, and Regent Whitelaw Reid was elected vice-chancellor to succeed Bishop Doane.

A number of charter amendments of schools working under regents' charters were approved and permanent library charters were granted to the Binghamton public library, the Oneida public library, the Adams free library, and the Massena free library. Provisional library charters were granted the Collins and East Rockaway free libraries.

Dr. L. A. Martin, of Binghamton, was appointed a state medical examiner to succeed Dr. Willard N. Bell, of Ogdensburg and Dr. E. D. Ackerman, of Oneida, was appointed a state veterinary examiner, to succeed Dr. F. L. Kilbourne, of Kelloggsville.

